# Cortical disinhibition occurs in chronic neuropathic, but not in chronic nociceptive pain

**DOI:** 10.1186/1471-2202-11-73

**Published:** 2010-06-11

**Authors:** Peter Schwenkreis, Andrea Scherens, Anne-Kathrin Rönnau, Oliver Höffken, Martin Tegenthoff, Christoph Maier

**Affiliations:** 1 Department of Neurology, Ruhr-University Bochum, BG-Universitätsklinikum Bergmannsheil, Bürkle-de-la-Camp-Platz 1, 44789 Bochum, Germany; 2 Department of Pain Management, Ruhr-University Bochum, BG-Universitätsklinikum Bergmannsheil, Bürkle-de-la-Camp-Platz 1, 44789 Bochum, Germany

## Abstract

**Background:**

The aim of this study was to examine the relationship between chronic neuropathic pain after incomplete peripheral nerve lesion, chronic nociceptive pain due to osteoarthritis, and the excitability of the motor cortex assessed by transcranial magnetic stimulation (TMS). Hence in 26 patients with neuropathic pain resulting from an isolated incomplete lesion of the median or ulnar nerve (neuralgia), 20 patients with painful osteoarthritis of the hand, and 14 healthy control subjects, the excitability of the motor cortex was tested using paired-pulse TMS to assess intracortical inhibition and facilitation. These excitability parameters were compared between groups, and the relationship between excitability parameters and clinical parameters was examined.

**Results:**

We found a significant reduction of intracortical inhibition in the hemisphere contralateral to the lesioned nerve in the neuralgia patients. Intracortical inhibition in the ipsilateral hemisphere of neuralgia patients and in both hemispheres of osteoarthritis patients did not significantly differ from the control group. Disinhibition was significantly more pronounced in neuralgia patients with moderate/severe pain intensity than in patients with mild pain intensity, whereas the relative compound motor action potential as a parameter of nerve injury severity did not correlate with the amount of disinhibition.

**Conclusions:**

Our results suggest a close relationship between motor cortex inhibition and chronic neuropathic pain in the neuralgia patients, which is independent from nerve injury severity. The lack of cortical disinhibition in patients with painful osteoarthritis points at differences in the pathophysiological processes of different chronic pain conditions with respect to the involvement of different brain circuitry.

## Background

Lesions of a motor or mixed peripheral nerve lead to short- and long-term reorganization of the motor cortex, as demonstrated in a large number of animal studies [1-3]. Similar motor cortex reorganization was shown to result from limb amputation in humans [4,5]. This motor cortex reorganization in amputees was linked to the occurrence and intensity of phantom limb pain [6], but was also observed in amputees without pain [7], as well as in patients with other chronic neuropathic pain conditions [8,9]. The exact relationship between peripheral deafferentation, neuropathic pain and motor cortex reorganization therefore remains still debatable. At least in its early phase, motor cortex reorganization is mainly based on functional changes of synaptic efficacy, involving the removal of local GABAergic inhibition [10], and long-term potentiation-like mechanisms [11]. These functional synaptic changes are thought to be reflected by changes in intracortical inhibition (ICI) and facilitation (ICF) assessed by paired-pulse transcranial magnetic stimulation (TMS), as previously demonstrated in limb amputees, who showed a reduced ICI and an enhanced ICF in the hemisphere contralateral to the amputation [12,13]. In patients with different chronic neuropathic pain syndromes of central and peripheral origin, ICI in the motor cortex was found to be reduced, whereas restoration of ICI by repetitive transcranial magnetic stimulation (rTMS) applied to the motor cortex was paralleled by a reduction in pain intensity, suggesting a close relationship between ICI and chronic neuropathic pain [14]. Similarly, patients with complex regional pain syndrome (CRPS) showed a reduced ICI, which was more pronounced in patients with higher pain intensity [15]. In these patients ICI was reduced not only in the contralateral, but also in the ipsilateral motor cortex, which points at differences in the underlying pathophysiological processes.

In the present study, we assessed ICI and ICF by paired-pulse TMS in patients with chronic neuropathic pain due to an incomplete peripheral nerve lesion, and with chronic nociceptive pain due to osteoarthritis. Our aim was to examine the relationship between chronic neuropathic pain of peripheral origin and cortical excitability changes in the motor cortex, and to assess possible differences between chronic neuropathic and nociceptive pain conditions.

## Results

### Group characteristics

There was no significant difference between the neuralgia group, the osteoarthritis group and the healthy controls with respect to the patients' age (F_2,57 _= 2.612, p = 0.082). Neuralgia patients did not significantly differ from osteoarthritis patients with respect to current (p = 0.173) and mean (p = 0.700) pain intensity, whereas maximum pain intensity was significantly higher in the osteoarthritis patients (p = 0.032; Cohen's d = 0.73). Duration of pain did not significantly differ between both groups (p = 0.788).

### Intracortical inhibition (ICI)

**Figure 1:**
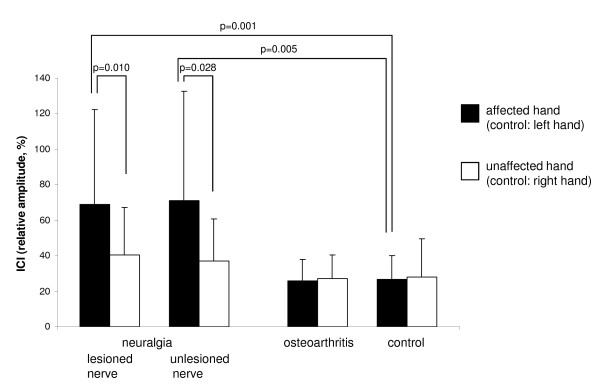
**Intracortical inhibition (ICI)**. Mean ICI (relative amplitude, expressed in %) in the neuralgia group, the osteoarthritis group and the control group. For the neuralgia group, results obtained from a muscle supplied by the lesioned nerve and results obtained from a muscle supplied by the unlesioned nerve are shown. Significant p values of post-hoc t-test are shown. Error bars indicate standard deviation.

Comparing ICI between the three groups, and looking at the recordings taken from the muscle supplied by the lesioned nerve in the neuralgia group (abductor pollicis brevis, APB, in median nerve lesions; adductor digiti minimi, ADM, in ulnar nerve lesions), there was a significant "group" effect (F_2,57 _= 10.433, p < 0.001), and a significant interaction between "side" and "group" (F_2,57 _= 4.560, p = 0.015), whereas the factor "side" did not reach significance (F_1,57 _= 3.140, p = 0.082). Post hoc analysis revealed a significantly reduced ICI at the neuralgia patients' affected side (relative amplitude 69.1 ± 53.4%) as compared to their unaffected side (relative amplitude 40.7 ± 26.4%, p = 0.010, Cohen's d = 0.67), whereas no significant side-to-side differences were observed in the osteoarthritis patients and the control group. ICI did not significantly differ between the osteoarthritis group and the control group. However, ICI was significantly reduced at the neuralgia patients' affected side as compared to the controls' left hand (relative amplitude 26.9 ± 13.0%, p = 0.001, Cohen's d = 1.09), whereas no significant difference was seen between the neuralgia patients' unaffected side and the controls' right hand (relative amplitude 27.8 ± 21.8%, p = 0.128) (Figure 1).

Including the results obtained from the muscle supplied by the unlesioned nerve in the neuralgia group in the statistical analysis (APB in ulnar nerve lesions; ADM in median nerve lesions), similar results were obtained: ANOVA also revealed a significant "group" effect (F_2,51 _= 8.949, p < 0.001), and a significant interaction between "side" and "group" (F_2,51 _= 4.356, p = 0.018), whereas the factor "side" did not reach significance (F_1,51 _= 3.387, p = 0.072). Again, post hoc testing revealed a significantly reduced ICI at the neuralgia patients' affected side (71.0% ± 61.8%) in comparison to their unaffected side (37.2% ± 23.7%, p = 0.028, Cohen's d = 0.72), but also in comparison to the controls' left hand (p = 0.005, Cohen's d = 0.99), whereas no significant difference was seen between the neuralgia patients' unaffected side and the controls' right hand (p = 0.253) (Figure 1).

### Intracortical facilitation (ICF)

**Figure 2:**
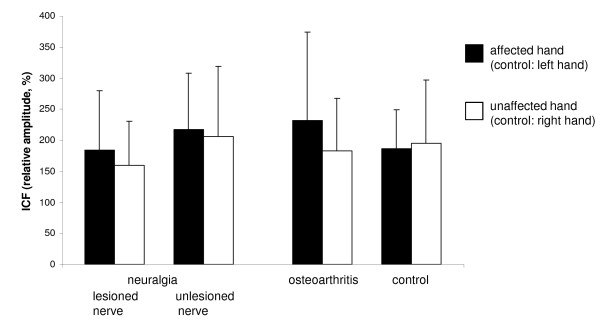
**Intracortical facilitation (ICF)**. Mean ICF (relative amplitude, expressed in %) in the neuralgia group, the osteoarthritis group and the control group. For the neuralgia group, results obtained from a muscle supplied by the lesioned nerve and results obtained from a muscle supplied by the unlesioned nerve are shown. Note that there are no significant differences between groups. Error bars indicate standard deviation.

Including the neuralgia group results obtained from the muscle supplied by the lesioned nerve in the statistical analysis (APB in median nerve lesions, ADM in ulnar nerve lesions), there was no significant effect of the factor "side" (F_1,57 _= 1.846, p = 0.180) or the factor "group" (F_2,57 _= 1.203, p = 0.308), and also no significant interaction between both factors (F_2,57 _= 1.038, p = 0.361). Similar results were obtained when the values obtained from the muscle supplied by the unlesioned nerve (APB in ulnar nerve lesions, ADM in median nerve lesions) were included in the analysis (F_1,51 _= 0.972, p = 0.329 for the factor "side", F_2,51 _= 0.290, p = 0.750 for the factor "group", F_2,51 _= 0.947, p = 0.395 for the interaction) (Figure 2).

### MEP amplitudes after single test stimuli

In the paired pulse TMS-paradigm used in this study, the intensity of the test stimulus (TS) was adjusted to evoke a MEP of approximately 1 mV. Therefore, a side-to-side-difference or a between-group-difference for the MEP amplitudes after single test stimuli should be excluded. This was confirmed by statistical testing including the results from the muscle supplied by the lesioned nerve (APB in median nerve lesions, ADM in ulnar nerve lesions) (F_1,57 _= 1.433, p = 0.236 for the factor "side", F_2,57 _= 1.737, p = 0.185 for the factor "group", F_2,57 _= 0.649, p = 0.526 for the interaction) as well as from the muscle supplied by the unlesioned nerve (APB in ulnar nerve lesions, ADM in median nerve lesions) in the neuralgia group (F_1,51 _= 1.487, p = 0.228 for the factor "side", F_2,51 _= 0.475, p = 0.625 for the factor "group", F_2,51 _= 0.068, p = 0.934 for the interaction).

### Test stimulus intensity

Looking at the TS intensity relative to the motor threshold (MT), and including the neuralgia group results obtained from the muscle supplied by the lesioned nerve (APB in median nerve lesions, ADM in ulnar nerve lesions) in the statistical analysis, there was no significant effect of the factor "side" (F_1,57 _= 1.102, p = 0.298) or the factor "group" (F_2,57 _= 1.199, p = 0.309), and also no significant interaction between both factors (F_2,57 _= 0.709, p = 0.497). Relative TS intensity was 124.0 ± 5.5% of MT at the affected and 123.6 ± 6.1% at the unaffected side in the neuralgia patients, 121.1 ± 6.7% at the affected and 122.3 ± 5.3% at the unaffected side in the osteoarthritis patients, and 121.2 ± 5.2% at the left hand and 122.9 ± 5.8% at the right hand in the control group.

Including the results obtained from the muscle supplied by the unlesioned nerve (APB in ulnar nerve lesions, ADM in median nerve lesions) in the analysis for the neuralgia group, no significant effect of the factor "side" (F_1,51 _= 0.829, p = 0.367), "group" ((F_2,51 _= 0.720, p = 0.491) or the interaction between "side" and "group" (F_2,51 _= 0.534, p = 0.589) was detected either. Relative TS intensity was 123.7 ± 7.1% of MT at the affected and 123.3 ± 5.8% at the unaffected side in the neuralgia patients.

### Motor threshold

There was no significant effect of one of the factors, neither when the results from the muscle supplied by the lesioned nerve (APB in median nerve lesions, ADM in ulnar nerve lesions) were included in the analysis (F_1,57 _= 1.228, p = 0.272 for the factor "side", F_2,57 _= 1.313, p = 0.277 for the factor "group", F_2,57 _= 0.229, p = 0.796 for the interaction), nor when the results from the muscle supplied by the unlesioned nerve (APB in ulnar nerve lesions, ADM in median nerve lesions) were included (F_1,51 _= 1.991, p = 0.164 for the factor "side", F_2,51 _= 1.448, p = 0.244 for the factor "group", F_2,51 _= 0.181, p = 0.835 for the interaction). MT in the neuralgia group was 55.5 ± 10.2% at the affected and 55.0 ± 10.6% at the unaffected side when recordings were drawn from a muscle supplied by the lesioned nerve, and 56.0 ± 8.8% at the affected and 54.5 ± 9.4% at the unaffected side when recordings were drawn from a muscle supplied by the unlesioned nerve. In the osteoarthritis group, MT was 52.5 ± 9.7% at the affected and 51.8 ± 10.0% at the unaffected side, and in the control subjects 58.2 ± 9.7% at the left and 55.9 ± 9.4% at the right hand.

In the neuralgia group, comparison between MT in the muscle supplied by the lesioned and the muscle supplied by the unlesioned nerve at the affected side did not reveal a significant difference (p = 0.450).

### Relationship between ICI and clinical parameters in neuralgia patients

In order to further differentiate between the repercussion of neuropathic pain and the repercussion of peripheral nerve lesion on ICI, patients with peripheral nerve lesion were divided into two separate groups according to their current pain intensity as assessed by numeric rating scale (NRS). Neuralgia patients with NRS≥4 were considered to have moderate/severe pain, patients with NRS<4 to have mild pain [16,17]. Two patients with poor German language competence were excluded. When the recordings were drawn from a muscle supplied by the lesioned nerve (APB in median nerve lesions, ADM in ulnar nerve lesions), motor cortex disinhibition was significantly more pronounced in the moderate/severe pain patients (83.2% ± 60.8%) than in the mild pain patients (41.7% ± 18.9%, p = 0.019, Cohen's d = 0.92). Recordings from a muscle supplied by the unlesioned nerve (APB in ulnar nerve lesions, ADM in median nerve lesions) revealed a similar tendency (83.6% ± 72.9% vs. 39.3% ± 20.3%), which however failed to reach statistical significance (p = 0.063). Further subgroup analysis did not reveal a significant difference in ICI between neuralgia patients with or without hypesthesia, dysesthesia, mechanical allodynia or muscle paresis. ICI did not differ between patients with lesions of the median and ulnar nerve, neither when considering the recordings from a muscle supplied by the lesioned nerve, nor by the unlesioned nerve.

There was no significant correlation between ICI and the relative amplitude of the compound muscle action potential (CMAP) after supramaximal electrical stimulation of the lesioned nerve at the wrist (expressed as a percentage of the CMAP of the contralateral homologous nerve), neither when the recordings were drawn from a muscle supplied by the lesioned nerve (r=-0.271, p = 0.181), nor when the recordings were drawn from a muscle supplied by the unlesioned nerve (r = 0.005, p = 0.984). There was no significant correlation between ICI and the pain duration either.

## Discussion

Our main finding was a significant reduction of ICI in the motor cortex contralateral to the affected hand in neuralgia patients, whereas painful osteoarthritis patients showed no abnormalities of ICI with respect to an age-matched control group.

In patients with entrapment of the median nerve at the carpal tunnel, which constituted a subgroup of patients in our study, it was previously shown that the chronic pathological modification of peripheral sensorimotor inputs leads to an enhanced excitability and a reorganization of the somatotopic map in the contralateral primary somatosensory cortex (S1) [18-21]. Similar plastic changes were shown to occur rapidly after a transient deafferentation induced by an anesthetic nerve bloc, being therefore more likely based on functional synaptic mechanisms than on axonal sprouting and the formation of new synapses [22,23]. The presence of similar changes in cortical excitability and cortical map somatotopy in the primary motor cortex (M1) of these patients has not been studied so far. However, our results strongly suggest that the reorganization previously observed in S1 is paralleled by similar changes in M1 of patients with incomplete peripheral nerve lesions.

Our results in neuralgia patients resemble the changes of ICI observed in the contralateral motor cortex of patients with upper or lower limb amputation [12,13]. Given the fact that motor cortex disinhibition is also present in patients with limb amputation who do not suffer from chronic pain [12], it might be argued that the disinhibition is more closely related to the peripheral nerve lesion rather than to the chronic neuropathic pain. However, there are a number of arguments, which favor the chronic neuropathic pain as the strongest determinant of the reduced inhibition in our study: First, dividing neuralgia patients into two subgroups according to their current pain intensity revealed a significantly more pronounced motor cortex disinhibition in the moderate/severe pain (NRS≥4) as compared to the mild pain patients. Second, the relative CMAP amplitude as an indicator of nerve injury severity did not correlate with ICI. In addition, ICI did not significantly differ between neuralgia patients with or without muscle paresis, indicating that cortical disinhibition was also independent from motor impairment. Third, there is evidence from various studies in animal models that an altered cortical excitation including reduction of GABA mediated inhibitory transmission is a crucial pathophysiological mechanism in chronic pain [24]. These excitability changes mainly have been demonstrated for the anterior cingulate cortex (ACC), but imaging studies revealed a close functional relationship between ACC and M1 in chronic neuropathic pain conditions [25,26]. Finally, in an interventional study using rTMS to enhance ICI in M1 of patients with chronic neuropathic pain of different central or peripheral origin, increased intracortical inhibition was accompanied by reduced pain intensity, emphasizing also the importance of the relationship between motor cortex inhibition and chronic neuropathic pain [14].

In our study, the reduced ICI was not restricted to recordings drawn from a muscle supplied by the affected nerve, but was also present when recordings were drawn from a muscle supplied by an unaffected nerve, indicating a more widespread lack of inhibition in the hand area of the hemisphere contralateral to the lesioned nerve. This finding points at reorganizational processes in areas adjacent to the cortical representation of the lesioned nerve. It is in line with findings in patients with upper limb amputation, who also exhibited reorganization in the cortical representation of proximal stump muscles [7,27]. Besides, it offers a possible explanation for recent findings in patients with chronic neuropathic pain, showing that rTMS applied to a motor cortical area adjacent to the representation of the painful zone is even more effective in pain relief than rTMS applied to the motor cortical area corresponding to the painful zone itself [28].

As a possible methodological problem, the MEP responses recorded from the APB muscle might have been contaminated by responses from ulnar muscles of the thenar eminence, and therefore contain information from muscles supplied by the lesioned as well as from muscles supplied by the unlesioned nerve. However, since ICI did not significantly differ between patients with median and ulnar nerve lesions, this might not have influenced our results.

In previous studies, it has been shown that the intensity of the TS is critical for the assessment of ICI, and that differences in TS intensities might confound ICI comparisons [29,30]. Especially, this would have been critical in neuralgia patients, if the TS intensity required to achieve a MEP of approximately 1 mV had been elevated due to the peripheral nerve lesion. However, an elevated TS intensity in the neuralgia group due to the peripheral nerve lesion as a potential confounding factor was excluded, since analysis of TS intensities relative to MT revealed no significant difference within- or between-groups. In addition, MT was similar in all groups, including both the muscle supplied by the lesioned and the muscle supplied by the unlesioned nerve in the neuralgia group. Finally, the fact that a similar ICI reduction was observed when recordings were drawn from the muscle supplied by the unlesioned nerve at the neuralgia patients' affected side strongly supports the assumption that assessment of ICI was not confounded when recordings were drawn from the muscle supplied by the lesioned nerve.

Motor cortex disinhibition was only seen in the neuralgia group, whereas the osteoarthritis patients did not differ from the control group. In osteoarthritis, pain is thought to arise as a consequence of physiological activation of primary nociceptive afferents by tissue-damaging stimuli and processing of this activity within the nociceptive system, whereas in chronic neuropathic pain such as neuralgia, pain arises by activity generated within the nociceptive system without physiological stimulation of nociceptors [31]. This different activation of the nociceptive system leads to differential plastic changes at the level of primary sensory and dorsal horn neurons [32]. Although it is conceivable that this different activation of the nociceptive system also leads to an involvement of different brain circuitry in distinct pain conditions, the extent of supraspinal reorganization and the factors driving the process are largely undiscovered [33]. A morphometric imaging study revealed differences in prefrontal and thalamic gray matter density as an indicator of different CNS neuroplasticity between patients with neuropathic and non-neuropathic chronic pain [34]. A number of functional imaging studies using fMRI or PET showed differences between neuropathic and nociceptive pain conditions with respect to the activated brain regions [33,35-38]. In addition, the pattern of intracortical inhibition in neuralgia patients did also differ from CRPS patients, who previously were shown to have reduced intracortial inhibition not only in the contralateral, but also in the ipsilateral motor cortex, suggesting a more widespread and bilateral reorganization of the motor system in CRPS as compared to neuralgia [8,15,39]. In this context, our present findings point at pathophysiological differences between chronic neuropathic and chronic nociceptive pain with respect to the involvement of central motor circuitry.

The finding of a pain-related motor cortex disinhibition in patients with chronic neuropathic, but not in chronic nociceptive pain may have an impact on our understanding of the therapeutical use of invasive or non-invasive motor cortex stimulation in chronic pain conditions. The restoration of defective ICI might be a crucial mechanism to achieve pain relief in chronic neuropathic pain, as previously demonstrated by means of rTMS applied to the motor cortex [14]. Interestingly, in this previous study only high-frequency rTMS led to an enhancement of ICI in patients with chronic neuropathic pain, although high-frequency rTMS generally is known to reduce ICI in healthy subjects [40], supporting the view that rTMS effects essentially depend on the state of cortical excitability before stimulation [14]. On the other hand, the lack of motor cortex disinhibition in patients with painful osteoarthritis as a chronic non-neuropathic pain condition might allow predicting a therapy failure of rTMS applied to the motor cortex in these patients.

## Conclusions

Our results suggest a close relationship between motor cortex inhibition and chronic neuropathic pain in the neuralgia patients, which is independent from nerve injury severity. The lack of cortical disinhibition in patients with painful osteoarthritis points at differences in the pathophysiological processes of different chronic pain conditions with respect to the involvement of different brain circuitry.

## Methods

### Subjects

We studied 26 patients (12 women, 14 men) with isolated incomplete lesion of the median (n = 16) or ulnar (n = 10) nerve as revealed by previous clinical and electroneurographical/electromyographical examination. In all patients, the relative amplitude of the distal compound muscle action potential (CMAP) after supramaximal electrical stimulation of the lesioned nerve at the wrist (expressed as a percentage of the CMAP of the contralateral homologous nerve) was determined, and served as a measure of nerve injury severity. All patients had a history of ongoing or intermittent neuropathic pain (neuralgia) due to the peripheral nerve lesion. Peripheral nerve lesion was due to trauma in 14 patients, due to entrapment of the median nerve at the carpal tunnel in ten patients, and due to entrapment of the ulnar nerve at the elbow in two patients. Patients with concomitant neurological disorders were excluded. The patients' age was between 24 and 77 years (mean 50.9 ± 11.7 years), and duration of the neuralgia between 3 and 190 months (mean 39.3 ± 44.8 months). The patients did not take any central acting drugs when participating in the study. Immediately before starting the TMS session, the current pain intensity was assessed, and the patients were also asked to retrospectively judge the mean and maximum ongoing pain intensity during the week prior to the TMS session using a numeric rating scale (NRS; values between 0 = "no pain" to 10 = "maximal pain"). Clinical details of the neuralgia patients are reported in Table 1.

**Table 1:** Clinical data of the neuralgia and painful osteoarthritis patients.

	Neuralgia	Painful osteoarthritis
N	26	20

Sex (women/men)	12/14	10/10

Age (years; mean±SD)	50.9±11.7	56.6±10.2

Affected side (left/right)	16/10	6/14

Cause and site of nerve lesion		

Carpal tunnel syndrome (median)	10	-

Traumatic, wrist (median)	6	-

Cubital tunnel syndrome (ulnar)	2	-

Traumatic, elbow (ulnar)	6	-

Traumatic, wrist (ulnar)	2	-

CMAP of the lesioned nerve (expressed as a percentage of the CMAP of the contralateral homologous nerve)	75.8±33.1	-

Duration of pain (months; mean±SD)	39.3±44.8	35.6±42.9

Pain intensity during previous week		

Mean (NRS; mean±SD)	4.8±1.8	5.0±1.5

Maximal (NRS; mean±SD)	6.0±2.2	7.4±1.6

Current pain intensity (NRS; mean±SD)	4.7±2.1	3.9±2.0

Number of patients with		

Hypesthesia	13 (50%)	0 (0%)

Dysesthesia	17 (65.4%)	0 (0%)

Mechanical allodynia	5 (19.2%)	0 (0%)

Muscle paresis	10 (38.5%)	0 (0%)

CMAP = compound muscle action potential; NRS = numeric rating scale; SD = standard deviation

In addition, 20 patients with unilateral painful primary degenerative osteoarthritis of the hand as a model of nociceptive, non-neuropathic pain (10 women, 10 men, aged between 41 and 76 years, mean 56.6 ± 10.2 years) were examined. A concomitant neurological disorder was excluded by clinical examination performed by an experienced neurologist (PS). Clinical details of the osteoarthritis patients are also reported in Table 1.

Results obtained in the patients were compared with results recorded from a control group of 14 right-handed healthy volunteers (8 women, 6 men, aged between 35 and 79 years, mean 58.8 ± 12.7 years). All subjects participating in the study gave their informed consent. The study was approved by the ethical committee of the Ruhr-University Bochum.

### Transcranial magnetic stimulation

TMS was performed using a bistim module, which was connected to two Magstim 200 stimulators (Magstim Co., Whitland, Dyfed, UK). The stimuli were applied through a circular coil (outer diameter 14 cm) positioned over the vertex with the current flowing anticlockwise in the coil in order to activate predominantly the left hemisphere, and clockwise in order to activate predominantly the right hemisphere. Earlier studies had shown that focal and circular coils elicited comparable results in paired-pulse studies [41]. While stimulating the contralateral hemisphere, recordings were taken with Ag-AgCl surface electrodes from the abductor pollicis brevis (APB) and from the abductor digiti minimi (ADM) muscle consecutively on both sides in the neuralgia patients, i.e., from a muscle supplied by the lesioned nerve (APB in median nerve lesions, ADM in ulnar nerve lesions), and from a muscle supplied by an unlesioned nerve (APB in ulnar nerve lesions, ADM in median nerve lesions). Six patients refused to participate in the whole experimental procedure, therefore recordings were only obtained from the muscle supplied by the lesioned nerve. In the osteoarthritis patients as well as in the control group, recordings were taken from the first dorsal interosseus (FDI) muscle. Previous paired-pulse TMS studies had revealed comparable results when recordings were obtained from different small hand muscles, including APB, ADM and FDI muscle [42,43]. The signals were recorded with a sampling rate of 5 kHz, and amplified with a bandpass of 20 Hz-3 kHz, a sweep duration of 10-50 ms/div and a gain of 0.1-1 mV/div. They were stored on an EMG machine (Neuropack 8, Nihon Kohden, Tokyo, Japan) for further analysis. Motor threshold (MT) was determined at rest to the nearest 1% of the stimulator output, and was defined as the minimum intensity that produced five motor evoked potentials >50 μV out of 10 trials [44]. The cortico-cortical excitability (intracortical inhibition and facilitation) was tested in the resting muscle by a paired-pulse paradigm [45]. The second stimulus (test stimulus, TS) was adjusted to evoke a motor evoked potential (MEP) of approximately 1.0 mV, the conditioning stimulus (CS) was set at 80% of the individual MT. MT, intensity of CS and intensity of TS were individually adjusted to each muscle in each patient. For paired-pulses, the interstimulus intervals (ISI) 2, 4, 10, and 15 ms were chosen. For each interval, at least eight responses were collected. The paired pulses were mixed with a total number of 32 suprathreshold single control stimuli, using the same stimulation intensity as for the second (test) stimulus. After the session, the amplitudes of all MEP responses were manually measured, and mean MEP amplitudes were calculated for each interstimulus interval as well as for the control condition. Then for each interstimulus interval the amplitude ratio of the mean conditioned MEP to the mean control MEP was calculated. Parameters of intracortical inhibition (ICI) and of intracortical facilitation (ICF) were defined as the averages of the MEP ratios obtained at inhibitory interstimulus intervals of 2 and 4 ms, and at facilitatory intervals of 10 and 15 ms [46]. For all recordings, subjects were given audio-visual feedback at high gain to assist complete muscle relaxation. If EMG activity became apparent during data collection, including a 10 ms pre-stimulus interval, responses were rejected.

To control for a possible influence of TS intensity on results of ICI and ICF, TS intensity (which had been adjusted to achieve an unconditioned MEP of approximately 1 mV) relative to MT was calculated and compared between groups.

### Statistical analysis

To analyze excitability parameters, results obtained at the neuralgia patients' affected side from the muscle supplied by the lesioned nerve were considered separately from results obtained from the muscle supplied by the unlesioned nerve. An ANOVA for repeated measurements was calculated, with "side" (affected vs. unaffected) as within-subjects factor, and "group" (neuralgia, osteoarthritis or control) as between-subjects factor, to analyze the different parameters. In the control group, the left hand was defined as the affected side, and the right hand as the unaffected side for statistical purposes. Greenhouse-Geisser procedure was used with epsilon-corrected degrees of freedom, if data showed significant deviations from sphericity. Paired and unpaired t-tests were used for post-hoc analysis if the ANOVA revealed a significant effect for one of the factors, or a significant interaction between factors. Single-factorial ANOVA was used to compare patients' age between groups, and unpaired t-tests to compare current, mean and maximum pain intensity as well as pain duration between neuralgia and osteoarthritis patients. Unpaired t-tests were also used to compare excitability parameters between subgroups of neuralgia patients with or without hypesthesia, dysesthesia, mechanical allodynia or muscle paresis, between neuralgia patients with lesions of the median or ulnar nerve, and between neuralgia patients with moderate/severe (NRS≥4) or mild (NRS<4) current pain intensity. A paired t-test was used to compare MT determined in both a muscle supplied by the lesioned nerve and a muscle supplied by the unlesioned nerve in neuralgia patients. Additionally, Cohen's d as an indicator of effect size was calculated if t-tests yielded a significant p value. Cohen's d is generally interpreted as follows: ≥1.0 very large; ≥0.8 large; ≥0.5 moderate; 0.2-0.4 small [47].

Pearson's correlation coefficient was calculated in order to detect a possible relationship between excitability parameters and pain duration, as well as between excitability parameters and the relative CMAP amplitude as a parameter of peripheral nerve injury severity.

For all statistical tests, the SPSS 14.0 software package (SPSS software, Munich, Germany) was used, and significance was assumed at the 0.05 level.

## Authors' contributions

PS, AKR and OH conducted the TMS experiments, performed the statistical analysis and drafted the manuscript. AS, MT and CM participated in the design and coordination of the study, and in the discussion of the results. All authors read and approved the final manuscript.
